# Baicalin Improves Skeletal Muscle Atrophy by Attenuating DRP-1-Mediated Mitochondrial Fission in Aged Mice

**DOI:** 10.3390/muscles4030035

**Published:** 2025-08-19

**Authors:** Hla Myat Mo Mo, Jong Han Lee

**Affiliations:** Department of Aquatic Life Medicine, Hanseo University, Seosan 31962, Republic of Korea; nicholelynn7@gmail.com

**Keywords:** sarcopenia, muscle atrophy, baicalin, inflammation, mitochondria fission, apoptosis

## Abstract

Baicalin is a natural flavonoid that has anti-apoptotic and anti-inflammatory effects. It shows some beneficial effects on muscle atrophy. However, its effects on age-related muscle atrophy are poorly understood. In this paper, we investigated whether baicalin exerts protective effect against skeletal muscle atrophy and its underlying mechanisms in aged mice using the grip strength test, histological analysis, and Western blots. Baicalin increased total muscle mass and strength in aged mice. Consistently, the cross-sectional area of quadriceps (QD) muscle significantly increased in both baicalin-administrated groups. Moreover, baicalin induced a shift in muscle fiber size distribution toward large fibers in both groups of mice. Expression levels of muscle atrophic factors, such as myostatin (MSTN) and atrogin-1, as well as pro-inflammatory cytokines, including tumor necrosis factor-alpha (TNF-α) and interleukin-6 (IL-6), were elevated in aged mice, but these increases were reduced by baicalin. While mitochondrial fission regulator, dynamin-related protein 1 (DRP-1), and apoptosis-related protein (apoptotic protease activating factor 1 (Apaf-1)) expressions were higher in aged mice than young mice, and their expression were downregulated following baicalin administration. The comprehensive results of this study suggest that baicalin provides beneficial effects on the treatment of sarcopenia not only by suppressing muscle atrophic factor expression and inflammation but also attenuating DRP-1-mediated mitochondrial fission and apoptosis.

## 1. Introduction

Sarcopenia is the age-related progressive and degenerative loss of skeletal muscle mass, strength, and function [1,2]. Its cause is widely considered multifactorial, involving hormonal changes, increased inflammation, decreased physical activity, and other metabolic changes [3]. Sarcopenia threatens the independent living of older adults, imposes financial burdens, and strains social systems and public health institutions [4,5]. In addition, it increased morbidity and mortality, highlighting its role as a critical factor in adverse health outcomes among the elderly [6]. Despite its widespread impact and growing recognition, there are currently no specific drugs approved by the Food and Drug Administration (FDA) to treat sarcopenia. The current management of sarcopenia primarily focuses on non-pharmacological approaches, such as resistance exercise training, nutritional supplementation, as well as lifestyle modifications aimed at preserving muscle mass and function [7,8].

Several molecular mechanisms have contributed to the occurrence of sarcopenia [1]. The well-known primary molecular mechanisms of sarcopenia include an imbalance between protein synthesis and degradation, inflammatory disturbances, and mitochondrial dysfunction [1,9]. The ubiquitin–proteasome (i.e., atrogin-1 and Muscle RING-finger protein-1 (MuRF-1)) and the autophagy pathways are mainly associated with protein degradation [1,10]. Myostatin (MSTN) is a well-known negative regulator of muscle mass. It activates Forkhead box O (FoxO) transcription factors, which in turn stimulate the expression of muscle-specific E3 ubiquitin ligases, such as atrogin-1 and MuRF-1, particularly under muscle atrophic conditions [11]. Pro-inflammatory cytokines (i.e., tumor necrosis factor-alpha [TNF-α] and interleukin-6 [IL-6]) levels are chronically elevated with aging. Nuclear factor kappa-light-chain enhancer of activated B cells (NF-κB), a catabolic transcription factor, is activated in response to cellular stress and inflammation, thereby inducing the expression of TNF-α and IL-6 in sarcopenia [12,13].

Maintaining mitochondrial metabolic function is essential for mitophagy and cell survival [14,15]. Mitochondrial function is mainly regulated by quality control mechanisms, including fusion and fission events under various physiological conditions [9]. Optic atrophy 1 (OPA-1) regulates inner mitochondrial membrane fusion, and its reduction impairs the oxidative phosphorylation of mitochondria, leading to mitochondrial fragmentation and contributing to the development of sarcopenia [16]. Dynamin-related protein 1 (DRP-1) serves as a critical regulator of mitochondrial fission in mammalian cells. DRP-1-dependent mitochondrial fission is a complex process that regulates cellular functions and organ dynamics. It is also involved in essential biological events such as development, apoptosis, acute organ injuries, as well as multiple pathological conditions [17,18]. One of the hallmarks of aging is dysregulation of mitochondrial function [19,20,21]. The disruption of mitochondrial network integrity in skeletal muscle is triggered by excessive mitochondrial fission, which leads to muscle weakness and muscle atrophy [19,22]. In addition, apoptotic protease activating factor 1 (Apaf-1) and caspase 3 play key roles in the intrinsic apoptosis pathway. Mitochondrial dysfunction triggers the release of cytochrome c into the cytoplasm, where it binds to Apaf-1 and procaspase-9 to form the apoptosome. This apoptosome then activates caspase-3 and induces apoptosis [23,24]. Therefore, enhancing mitochondrial function may help to attenuate age-related muscle atrophy.

Under cellular stress conditions, increased mitochondrial fission has been determined to be responsible for the elevation of reactive oxygen species (ROS), energy metabolism impairment, and release of pro-apoptotic factors [25]. In particular, dysfunction of DRP-1 is associated with the development of neuropathic pain [26], cardiac arrest [27], and ischemic injury in the heart and brain. Its suppression has been shown to mitigate disease states and improves survival [28]. A recent study indicates that unbalanced mitochondrial dynamics implicated in cachexia-induced muscle atrophy and restoring the mitochondrial function by mediating DRP-1 prevents muscle atrophy in cancer cachectic patients [29]. On the other hand, it has not been well studied on muscle atrophy with aging. Therefore, suppressing DRP-1-induced mitochondrial fission may be an interesting therapeutic approach to effectively treat age-related sarcopenia.

Baicalin is one of the major phenolic flavonoids from the root of the perennial herb *Scutellaria baicalensis*, which is known as classical Chinese herbal medicine [30,31]. It has attracted considerable scientific interest owing to its wide range of pharmacological activities, including anti-oxidative, anti-inflammatory, anti-viral, and anti-cancer properties. At the molecular level, baicalin possesses potential antioxidant properties by effectively scavenging free radicals and reducing oxidative stress. Moreover, it exhibits strong anti-inflammatory effects by modulating key signaling pathways involved in the inflammatory response [30,32]. Recently, it has been reported that baicalin has the effects to improve cardiac outcome and survival by regulating mitochondrial dynamics and attenuating ROS production in cardiac arrest-induced rats, and protects skeletal muscle injury by inhibiting the apoptotic cell death pathway [33,34]. However, it is not known whether baicalin plays a protective role against age-associated muscle atrophy by controlling mitochondrial fission.

In this study, we examined whether baicalin administration alleviates muscle atrophy in aged mice. Additionally, we sought to elucidate the underlying molecular mechanisms responsible for its effects, with a particular focus on the role of mitochondrial dynamics in age-related muscle atrophy. Our current results reveal that baicalin mitigates muscle atrophy and improves muscle strength in aged mice by reducing inflammation, attenuating DRP-1-mediated mitochondria fission, and inhibiting apoptosis.

## 2. Results

### 2.1. Baicalin-Treated Aged Mice Exhibit Higher Muscle Weight

**Figure 1:**
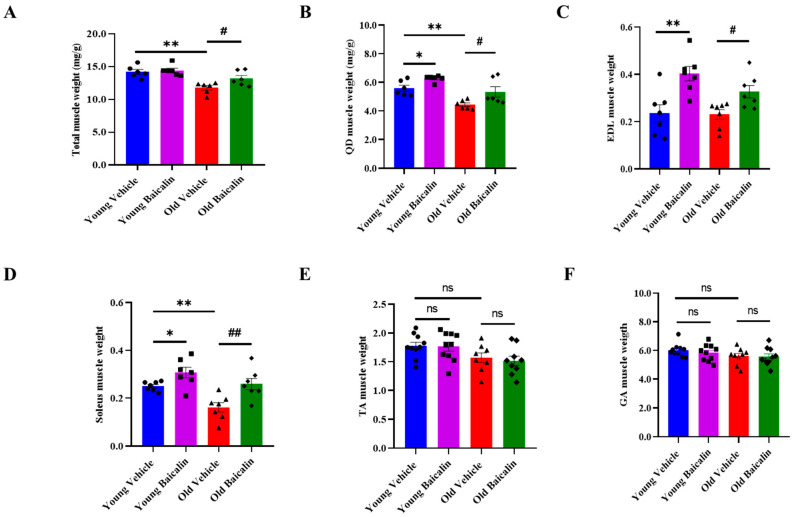
Baicalin-treated aged mice exhibit higher muscle weight. (**A**) Total muscle weight. (**B**) Muscle weights of the quadriceps (QD), (**C**) extensor digitorum longus (EDL), (**D**) soleus, (**E**) tibialis anterior (TA), and (**F**) gastrocnemius (GA). The muscle weights of the mice were normalized to the body weight (g). Total muscle weight represents the sum of all muscle weights such as QD, EDL soleus, TA, and GA. All values are expressed as the mean ± standard error. Significant differences are indicated as * *p* < 0.05 or ** *p* < 0.01 with vehicle young mice, ^#^ *p* < 0.05 or ^##^ *p* < 0.01 with vehicle old mice. ns: no significance. ● young vehicle, ■ young baicalin, ▲ old vehicle, ◆ old baicalin. *n* = 6–10/group.

Baicalin-treated aged mice exhibited slightly higher body weight (BW) than the vehicle group during the entire experimental period. However, there were no significant changes in both young and aged mice between the vehicle and baicalin groups (Supplementary Figure S1). Total muscle weight was remarkably lower in aged mice compared to that in young vehicle mice (Figure 1A). In contrast, it significantly increased following baicalin treatment in aged mice, but there was no difference in young mice group (Figure 1A). In vehicle-treated aged mice, the weights of all five muscle types were low (Figure 1B–F), with a particularly notable difference of 21% from 5.54 (mg/g) to 4.44 (mg/g) in the quadriceps (QD) and 36% from 0.25 (mg/g) to 0.16 (mg/g) in the soleus. However, baicalin-treated aged mice showed significantly higher muscle weight in the QD, extensor digitorum longus (EDL), and soleus compared to the vehicle group (Figure 1E,F). On the other hand, there was no difference in the muscle weight of the tibialis anterior (TA) and gastrocnemius (GA) between groups (Figure 1E,F).

### 2.2. Baicalin-Treated Aged Mice Exhibit Higher Muscle Strength

**Figure 2:**
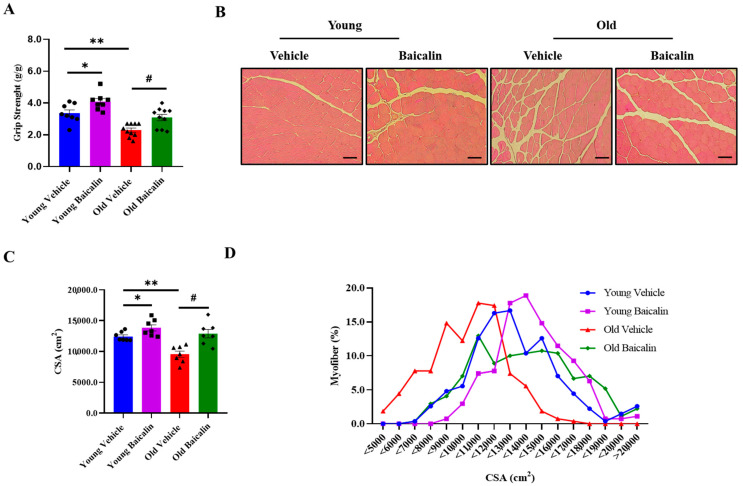
Baicalin-treated aged mice exhibit higher muscle strength and myofiber size. (**A**) Grip strength normalized to body weight (g). (**B**) Representative image of QD muscle tissue stained with hematoxylin and eosin (H&E). (**C**) Cross-sectional area (CSA) of QD muscle. (**D**) Muscle fiber size distribution. Scale bar = 200 μm. All values are expressed as the mean ± standard error. Significant differences are indicated as * *p* < 0.05 or ** *p* < 0.01 with vehicle young mice, ^#^ *p* < 0.01 with vehicle old mice. ● young vehicle, ■ young baicalin, ▲ old vehicle, ◆ old baicalin. *n* = 6–10/group.

The grip strength in aged mice (25.5-month-old) before baicalin treatment was remarkably lower than that in vehicle-treated young mice (5.5-month-old). On the other hand, baicalin treatment significantly enhanced grip strength in both young and aged mice (Figure 2A). Moreover, the grip strength observed in baicalin-treated mice was comparable to that of vehicle-treated young mice (Figure 2A). The cross-sectional area (CSA) of QD muscle fibers was smaller in vehicle-treated old mice relative to vehicle-treated young mice. However, the CSA of muscle fibers was greater with baicalin administration in both age groups of mice, and the increased CSA was positively correlated with grip strength (Figure 2B,C). In addition, the muscle fiber size distribution in baicalin-treated mice showed a significant predominance of larger fibers compared to vehicle-treated mice (Figure 2D).

### 2.3. Baicalin-Treated Aged Mice Exhibit Lower Expression Levels of Muscle Atrophic Factors and Inflammatory Cytokines

**Figure 3:**
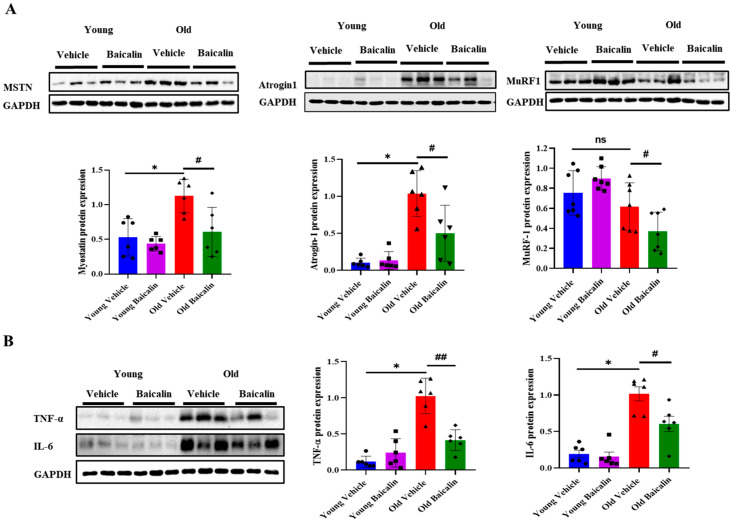
Baicalin decreases the expression of muscle atrophic factors and inflammatory cytokines in aged mice but no significant differences in young mice. (**A**) The protein level of MSTN, atrogin-1, and MuRF-1 in QD muscle. (**B**) The protein level of TNF-α and IL-6 in QD muscle. All values are expressed as the mean ± standard error. Significant differences are indicated as * *p* < 0.01 with vehicle young mice or ^#^ *p* < 0.05 or ^##^ *p* < 0.01 with vehicle old mice. MSTN, Myostatin; MuRF-1, Muscle ring-finger protein 1; TNF-α, Tumor necrosis factor alpha; IL-6, Interleukin-6. ns: no significance. ● young vehicle, ■ young baicalin, ▲ old vehicle, ◆ old baicalin. *n* = 6–7/group.

The expression of MSTN and its target gene atrogin-1 were significantly higher in aged mice compared to young mice. Baicalin treatment showed their low expression levels in aged mice, while their expression in young mice remained unchanged (Figure 3A). In contrast, the protein expression of MuRF-1 was lower in aged mice than in young mice, but baicalin in aged mice resulted in a further reduction (Figure 3A). Since aging causes muscle atrophy by activating catabolic systems through inflammatory cytokines [28], we next investigated whether baicalin inhibits increased inflammation. As would be expected, aged mice exhibited a higher expression of TNF-α and IL-6 than young mice, but baicalin attenuated the expression of both cytokines, as shown in Figure 3B. In addition, there was no difference in young mice between the vehicle and treatment groups (Figure 3B).

### 2.4. Baicalin-Treated Aged Mice Exhibit Lower Levels of Mitochondrial Dysfunction and Apoptosis

**Figure 4:**
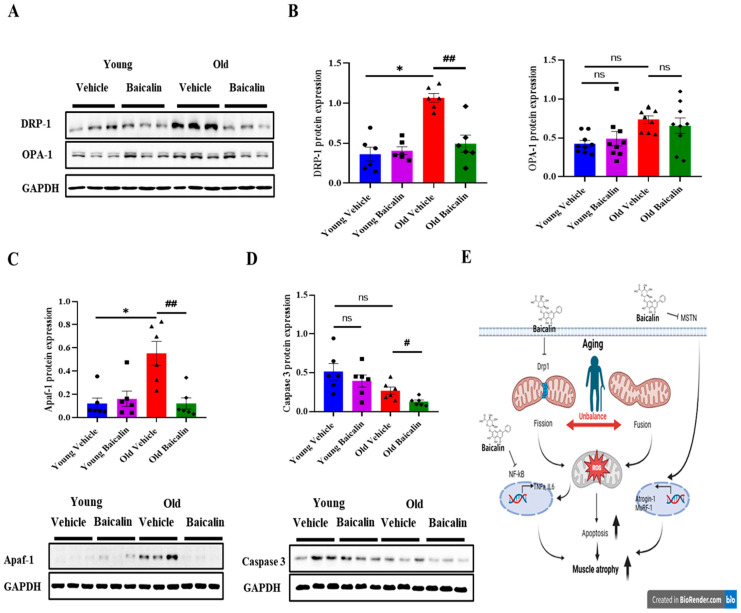
Baicalin suppresses apoptosis by regulating Drp-1 expression in aged mice. (**A**) The representative Western blot image level of DRP-1 and OPA-1 in QD muscle. (**B**) The protein level of DRP-1 and OPA-1 in QD muscle. (**C**) The protein level of Apaf-1 and (**D**) caspase 3 in QD muscle. (**E**) Schematic illustration of the proposed mechanism by which baicalin acts in muscle tissue. Our findings suggest that baicalin may modulate muscle atrophy in aged mice through three independent and interconnected signaling pathways: (1) Baicalin downregulates Drp-1 expression, thereby inhibiting mitochondrial fission. This inhibition leads to reduced mitochondrial ROS production and subsequent suppression of NF-κB signaling in muscle tissue. The NF-κB pathway promotes the expression of proinflammatory cytokines and muscle atrophy-related factors. (2) The elevation of proinflammatory cytokines simultaneously activates the myostatin signaling pathway, which in turn promotes the expression of muscle atrophy-related factors, particularly components of the muscle-specific E3 ubiquitin ligase system and (3) Mitochondrial dysfunction induces apoptotic signaling cascades thereby inducing muscle cell death. All values are expressed as the mean ± standard error. Significant differences are indicated as * *p* < 0.01 with vehicle young or ^#^ *p* < 0.05 or ^##^ *p* < 0.01 with vehicle old mice. DRP-1, Dynamin-related protein 1; OPA-1, Optic atrophy 1; Apaf-1, Apoptotic protease activating factor 1; ns: no significance. ● young vehicle, ■ young baicalin, ▲ old vehicle, ◆ old baicalin. *n* = 6–9/group.

Given that Drp-1-mediated mitochondrial fission contributes to the process of muscle atrophy [10,29], we further investigated whether baicalin inhibits the expression of mitochondrial fission-related protein DRP-1. The protein expression of DRP-1 in aged mice was significantly higher than young mice. However, baicalin administration resulted in lower levels in aged mice, but had no changes in young mice (Figure 4A,B). In contrast, the expression of mitochondrial fusion-related protein, OPA-1 was slightly higher in aged mice. However, there were no significant differences with baicalin, as shown in Figure 4A,B. Mitochondria dysfunction induces the expression of apoptosis-related proteins, which in turn leads to cell death. The expression of Apaf-1, a key component of the apoptosome complex, was significantly increased in aged mice compared to young mice. However, baicalin treatment did not alter Apaf-1 expression in young mice, with levels remaining comparable to untreated controls (Figure 4B). In addition, caspase-3 expression levels did not differ significantly between young and old mice. On the other hand, baicalin administration in aged mice led to a statistically significant reduction in caspase-3 expression (Figure 4B).

## 3. Discussion

A previous study reported that baicalin exhibits a wide range of pharmacological activities. These include potent anti-inflammatory effects, which help reduce chronic inflammation; strong antioxidant properties that combat oxidative stress; and the capacity to regulate mitochondrial function, thereby maintaining cellular energy homeostasis and promoting cell survival. Together, these properties contribute to baicalin’s therapeutic potential in various pathological conditions [30,31,32,33]. However, the efficacy of baicalin on age-related sarcopenia has not been explored yet. In the current study, we examined the role of baicalin in age-related muscle atrophy, as in sarcopenia. Our current study revealed that baicalin has significant beneficial effects on age-induced muscle atrophy by promoting increases in muscle mass and enhancing muscle function. Baicalin mechanically protects against muscle atrophy by suppressing inflammation, inhibiting the ubiquitin–proteasome system, alleviating mitochondrial dysfunction, and preventing apoptosis in aged mice. Notably, its inhibition of DRP-1-mediated mitochondrial fission plays a key role in mitigating mitochondrial dysfunction and apoptosis in skeletal muscle in aged mice.

Aging leads to a decline in total muscle mass, strength, and function [2,35]. For quantifying total muscle mass, we collected five distinct types of skeletal muscles, namely QD, EDL, soleus, TA, and GA from the lower limb. These all-muscle fiber types have their distinct fiber-type compositions and anatomical locations. In addition, their relatively large size and clear anatomical boundaries facilitate consistent dissection, allowing for accurate measurement of muscle weight and morphology. In the present study, we observed that the weights of all examined skeletal muscle types were significantly lower in aged mice compared to young mice. This age-related reduction in muscle mass is consistent with the findings reported in our previous study, further supporting the notion that aging is closely associated with progressive skeletal muscle atrophy [35]. On the other hand, baicalin treatment for 4 weeks (wks) led to a higher level of muscle mass in QD, EDL, and soleus muscle mass, along with a significant improvement in total muscle mass and grip strength in aged mice. Interestingly, in young mice, baicalin enhanced muscle strength without altering total muscle mass. These findings suggest that baicalin’s effects on muscle may be more pronounced under pathological conditions such as aging, potentially through mechanisms like improving age-related physiological alterations (e.g., chronic low-grade inflammation).

Although the loss of whole muscle mass and muscle fiber size may not be the only or main cause of sarcopenia, it nonetheless plays a substantial role in its progression [36]. The aging process is consistently accompanied by a marked decrease in the CSA of skeletal muscle fibers, which not only reflects quantitative losses in muscle mass but also signifies qualitative alterations in muscle composition and function [37,38]. These changes collectively contribute to the decline in muscle strength and functional capacity observed in elderly populations [36]. Consistently, our histological analysis of the QD muscles revealed that baicalin increased fiber size in both age group mice. Notably, aged mice exhibited a predominance of small-sized fibers (<10,000 μm^2^) compared to that of young mice. However, following baicalin administration, the muscle fiber size distribution shifted toward middle- and large-sized fibers, aligning with an increase in average CSA. Baicalin exhibited greater effects in aged mice than in young mice. Collectively, such changes in fiber size distribution indicate that baicalin promotes muscle cell differentiation and enhances muscle fiber thickness while preventing muscle atrophy, ultimately leading to an increased proportion of larger muscle fibers and consequently improving muscle mass and strength.

In older adults, a persistent, low-grade systemic inflammation—referred to as “inflammaging”—is linked to various age-related diseases, including sarcopenia [35,39]. The administration of IL-6 or TNF-α to rats has been shown to induce muscle breakdown, suggesting that inflammation contributes to the loss of muscle mass and strength with aging [40]. In addition, growing clinical evidence supports a strong link between chronic low-grade inflammation and the development of sarcopenia. Elevated circulating concentrations of pro-inflammatory cytokines, particularly IL-6 and TNF-α, have been consistently associated with reduced muscle mass and diminished muscle strength in older populations [41,42]. This relationship has been reinforced by meta-analytic study showing that higher levels of IL-6, TNF-α, and C-reactive protein are significantly correlated with lower grip strength, reduced knee extension force, as well as decreased skeletal muscle mass. These associations include diverse populations, such as community-dwelling older adults, nursing home residents, and individuals with chronic diseases, from North America, Europe, and Asia [43]. Furthermore, interventional studies show that reducing systemic inflammation by structured physical activity programs attenuates muscle loss and functional decline, highlighting inflammation as a modifiable target in the prevention and management of sarcopenia [44,45].

Muscle atrophy is primarily driven by two major protein degradation pathways: the ubiquitin–proteasome system and the autophagy–lysosome system, both regulated by transcription factors such as FoxO and NF-κB [46,47]. Inflammatory cytokines induce the upregulation of MSTN and NF-κB expression in elderly individuals, thereby promoting catabolic signaling pathways in skeletal muscle. The subsequent activation of MSTN signaling further stimulates FoxO-dependent MuRF-1 and atrogin-1 expression. Consistently, our data showed that pro-inflammatory cytokines such as TNF-α and IL-6, along with MSTN, were upregulated in the QD muscle of aged mice, leading to increased expression of atrogin-1, a key mediator of ubiquitin–proteasome system activation. However, these increases were reversed by baicalin administration. Interestingly, the effects of baicalin were more pronounced in aged mice compared to young mice. Taken together, these findings suggest that (i) baicalin can attenuate E3 ligase expression in sarcopenic conditions by inhibiting inflammatory pathways, and (ii) the greater efficacy in aged mice may be due to higher basal levels of inflammation in aged mice compared to young mice.

Mitochondrial dysfunction plays a crucial role in aging-related muscle atrophy [35], driven by an imbalance between mitochondrial fusion and fission [21,35,48]. The fusion process is regulated by GTPases such as mitofusin (Mfn) 1, Mfn2, and OPA-1, while the fission machinery includes proteins such as fission (FIS1) and DRP-1 [16,19,49]. Impaired mitochondria dynamics activates the ubiquitin–proteasome system and apoptosis signaling, leading to muscle atrophy [9,35,50]. Fragmented mitochondria frequently appear in the early stages of apoptosis, accompanied by increased mitochondrial outer membrane permeability, thereby leading to the release of pro-apoptotic factors such as AIF and cytochrome c [51,52]. Although controversial [50], DRP-1-mediated apoptosis is implicated in age-related muscle cell loss and dystrophin-dependent muscle degeneration [53]. In addition, Wojtysiak et al. found that capon breast muscles had significantly lower levels of intact desmin and dystrophin at 24 and 48 h postmortem than cockerels. This slower degradation in cockerel muscles may explain their smaller muscle fiber diameter at later postmortem times. Both studies indicate that the pivotal role of DRP-1-mediated apoptosis in muscle atrophy and dysfunction [54]. Our findings show that mitochondrial fission regulated by DRP-1 is upregulated in aged mice but significantly downregulated by baicalin. On the other hand, OPA-1 expression remained unchanged in both young and aged mice, suggesting that baicalin specifically targets fission-related mechanisms rather than fusion under our current experimental condition to maintain mitochondria function. The intrinsic apoptotic pathway is initiated by the formation of the cytosolic apoptosome complex, comprising cytochrome c, Apaf-1, and procaspase-9, leading to caspase-9 activation and subsequent caspase-3 activation [23,24]. Baicalin has a protective effect against skeletal muscle injury by ameliorating apoptosis in cellular models [34]. Consistently, our data demonstrated that Apaf-1 protein levels were elevated in aged mice but significantly reduced following baicalin treatment. This reduction was accompanied by a decrease in the expression of caspase-3, a downstream effector of Apaf-1. These findings confirm that baicalin prevents age-related muscle atrophy via inhibiting the DRP-1-mediated apoptotic signaling pathway.

## 4. Materials and Methods

### 4.1. Animal Experiment

Young (4 months old) and old (24 months old) male C57BL/6 mice were obtained from Central Lab. Animal Inc. (Seoul, Korea). The mice were maintained as previously described [35]. Briefly, the animals were housed in a controlled environment where the temperature was maintained at approximately 23 ± 1 °C to ensure thermal comfort and minimize stress. They were kept under a strict 12-h light and 12-h dark cycle to simulate natural circadian rhythms. The relative humidity in the housing facility was carefully regulated at 50 ± 5%. Throughout the study period, the animals had unrestricted access to a standard laboratory diet (AIN-93G, Research Diets Inc., Foster Lane Flemington, NJ, USA; designated as the control diet) and clean drinking water, allowing for ad libitum feeding and hydration. The nutritional composition used for the current study was as follows: protein, 18.1%; fat, 7.1%; fiber, 4.8%; ash, 2.2%; moisture, less than 10%; and carbohydrates, 59.3%. The total caloric content was 3.74 kcal per gram. Mice were divided randomly into four groups: (1) Young-vehicle (*n* = 10), (2) Young-baicalin group, (*n* = 10), (3) Old-vehicle (*n* = 10), and (4) Old-baicalin (*n* = 10). Baicalin was dissolved in 5% dimethyl sulfoxide (DMSO) in distilled water immediately before administration and 100 mg/kg was orally administered once daily for 4 wks. The vehicle group received 5% DMSO in distilled water only. BW was assessed at the same time every morning. All experiments and research procedures are proceeded according to the protocols (LCDI-2022-0139, 22 December 2022) approved by the Institutional Animal Care and Use Committee at Gachon University.

### 4.2. Grip Strength Test

The grip strength test was performed by a trained researcher who was blinded to the experimental groups to ensure consistency and minimize bias. The test was conducted after 4 wks of baicalin treatment with a grip strength meter (BIO-G53, BIOSEB, Pinellas Park, FL, USA), as previously mentioned in our published paper [35]. In short, young (5.5-month-old) or old (25.5-month-old) mice were carefully lifted and held by the base of their tails, allowing their forelimbs and hindlimbs to naturally grasp onto a horizontal metal mesh. To assess limb strength, each mouse was slowly and steadily pulled backward by the tail while maintaining its body in a horizontal position parallel to the surface of the testing table. This continued until the mouse released its grip from the mesh, at which point the peak resistance force exerted by the limbs was recorded in grams (g) using a calibrated force measurement device. Each mouse underwent three consecutive trials under the same conditions, and the mean value of the three measurements was calculated and used for statistical analysis to ensure reliability and reduce variability.

### 4.3. Collection of Biological Samples

Following a 4-week period of treatment, the mice were fasted for 4 h (h) in the morning, and the body weights were carefully measured. Briefly, the mice were first anesthetized with 1–2% isoflurane. Right after harvesting the blood samples, the mice were humanly sacrificed by CO_2_ inhalation in accordance with the guideline of the institutional Animal Care and Use Committee. Five types of skeletal muscles (QD, EDL, soleus, TA, GA) were collected and the muscle weights were measured, and all samples were stored at −80 °C for further analysis. For histological analysis, some parts of skeletal muscle tissue were cut and immediately submerged in 10% natural buffer formalin (NBF).

### 4.4. Histochemical Staining

Formalin-fixed QD muscle tissues were submitted to the Histopathology Core Facility of the Lee Gil Ya Cancer and Diabetes Institute for paraffin embedding and sectioning. Tissues were fixed in 4% paraformaldehyde at 4 °C for 24 h, dehydrated through a graded ethanol series, cleared in xylene, and embedded in paraffin. The muscle blocks were embedded in a transverse (cross-sectional) orientation. Paraffin sections were cut at a thickness of 8 µm using a rotary microtome. Slides were stained with hematoxylin and eosin staining kit (ab245880, Abcam, Waltham, MA, USA). Briefly, tissue sections were deparaffinized in 100% xylene for 5 min, repeated twice, and then rehydrated through a graded ethanol series (100%, 95%, 80%, and 70%) for 2 min at each concentration. The slides were subsequently stained with hematoxylin for 2 min to visualize cell nuclei, followed by eosin staining for 2 min to highlight the cytoplasm and extracellular matrix. After staining, the sections were dehydrated through ascending ethanol concentrations, cleared in xylene, and mounted using a mounting medium. The stained tissues were observed under a light microscope (Olympus CKX53, OLYUMPUS CORPORATION, Tokyo, Japan). To analyze CSA of each muscle, at least thirty randomly selected non-overlapping fields of view were evaluated at 200× magnification for each slide and analyzed using the Image J 1.54p software.

### 4.5. Western Blot

Western blotting was conducted as previously described [35]. Briefly, the total proteins were extracted from QD muscle tissue with Radioimmunoprecipitation (RIPA) lysis buffer (R4100-010, GenDEPOT, Katy, TX, USA) containing 2% protease inhibitor mixture 100X (P3300-005, GenDEPOT, USA). The protein concentration was determined using the Pierce BCA Protein Assay Kit (WJ335000, Thermo Scientific, Waltham, MA, USA). Approximately 20 µg of the lysates was electrophoresed and transferred to the nitrile cellulose transfer membrane (1060004-Premium 0.2 um NC, Amersham Protran, Freiburg, Germany). Membranes were blocked with 5% nonfat milk in tris-buffered saline with 0.1% Tween 20 (TBST) and incubated with primary antibodies overnight at 4 °C. On the following day, the membrane was washed with TBST solution and incubated for 1 h at room temperature with the appropriate Horseradish Peroxidase (HRP)-conjugated secondary antibodies: anti-rabbit IgG (A120-101P, Bethyl Laboratories Inc., Montgomery, TX, USA) and anti-mouse IgG (A90-116P, Bethyl Laboratories Inc., USA). The same membrane was then stripped with 15 mL of stripping buffer (CSB500, Western Blot Stripping Buffer, LPS Solution, Seoul, Korea) at 37 °C for 15–30 min and reprobed with anti-Glyceraldehyde-3-phosphate dehydrogenase (GAPDH) antibody. Protein bands were visualized using an enhanced chemiluminescence kit (WBKLS0500, Immobilon Western Chemiluminescent HRP Substrate, Merck Millipore, Burlington, MA, USA) and detected with a molecular imager (Bio-Rad, Hercules, CA, USA) equipped with the Image Lab 5.2 software. The following antibodies were used: anti-myostatin (MSTN) (ab203076, Abcam, USA); anti-muscle RING-finger protein-1 (MuRF-1) (sc-398608, Santa Cruz Biotechnology, Dallas, TX, USA); anti-atrogin-1 (sc-166806, Santa Cruz Biotechnology); anti-optic atrophy 1 (OPA-1) (612606, BD Biosciences, San Jose, CA, USA); anti-tumor necrosis factor α (TNF-α) (sc-12744, Santa Cruz Biotechnology, Dallas, TX, USA); anti-interleukin-6 (IL-6) (sc-57315, Santa Cruz Biotechnology, Dallas, TX, USA); anti-DRP-1 (sc-271583, Santa Cruz Biotechnology, Dallas, TX, USA); anti-apoptotic protease activating factor 1 (Apaf-1) (sc-65891, Santa Cruz Biotechnology, Dallas, TX, USA); anti-caspase 3 (14220S, Cell Signaling Tech, Danvers, MA, USA); and anti-GADPH (sc-47724, Santa Cruz Biotechnology). The protein band quantification was measured using the ImageJ 1.54p software.

### 4.6. Statistical Analysis

All results are presented as the mean ± standard error. Statistical analyses were performed using a one-way or two-way analysis of variance (ANOVA), as appropriate, with the IBM SPSS Statistics 19 software (IBM Corp., Armonk, NY, USA), followed by Tukey’s multiple comparisons test or Student’s *t*-test for post hoc analysis. A two-way ANOVA was used to evaluate the main effects of age and treatment, as well as their interaction. Data normality and homogeneity of variances were assessed using the Shapiro–Wilk test and Levene’s test, respectively. A *p* value of < 0.05 was considered as statistically significance.

## 5. Conclusions

Our findings suggest that baicalin may play a regulatory role in the attenuation of muscle atrophy observed in aged mice by modulating three distinct but interrelated signaling pathways (Figure 4E): (1) Baicalin downregulates Drp-1 expression, thereby inhibiting mitochondrial fission. This inhibition leads to reduced mitochondrial ROS production and subsequent suppression of NF-κB signaling in muscle tissue. The NF-κB pathway promotes the expression of proinflammatory cytokines and muscle atrophy-related factors. (2) The elevation of proinflammatory cytokines simultaneously activates the myostatin signaling pathway, which in turn promotes the expression of muscle atrophy-related factors, particularly components of the muscle-specific E3 ubiquitin ligase system. (3) Mitochondrial dysfunction leads to the release of cytochrome c into the cytosol, which binds to Apaf-1 to form the apoptosome complex. This complex then activates initiator caspases, such as caspase-9, triggering downstream cascades that ultimately result in muscle cell apoptosis. Therefore, these findings highlight baicalin’s preventive effects against aging-induced muscle atrophy and underscore its potential as a therapeutic agent for the treatment of muscle atrophy.

## Data Availability

The data presented in this study are available upon request from the author.

## Supplementary Materials

The following supporting information can be downloaded at: https://www.mdpi.com/article/10.3390/muscles4030035/s1. Figure S1: Body weight change. Body weight was assessed daily for 4 weeks. Significant differences are indicated as * *p* < 0.05 with old vehicle mice.

## Abbreviations

FDA	Food and Drug Administration
QD	Quadriceps
MSTN	Myostatin
TNF-α	Tumor necrosis factor alpha
IL-6	Interleukin-6
DRP-1	Dynamin-related protein 1
Apaf-1	Apoptotic protease activating factor 1
MuRF-1	Muscle RING-finger protein-1
Myo D	Myoblast determination protein 1
FoxO	Forkhead box O
NF-κB	Nuclear factor kappa-light-chain-enhancer of activated B cells
OPA-1	Optic atrophy 1
ROS	Reactive oxygen species
BW	Body weight
EDL	Extensor digitorum longus
TA	Tibialis anterior
GA	Gastrocnemius
CSA	Cross-sectional area
Mfn	Mitofusin
FIS1	Fission
AIF	Apoptosis-inducing factor
NBF	Natural buffer formalin
DMSO	Dimethyl sulfoxide
RIPA	Radioimmunoprecipitation
SEM	Standard error mean
