# Supplementary material for: Baicalin Improves Skeletal Muscle Atrophy by Attenuating DRP-1-Mediated Mitochondrial Fission in Aged Mice
Source: Muscles. 2025 Aug 19;4(3):35. doi: 10.3390/muscles4030035 (PMC12371914; doi:10.3390/muscles4030035)
Supplement: Supplementary file 1 [file muscles-04-00035-s001.zip › muscles-3768279-supplementary.pptx]

## Slide 1
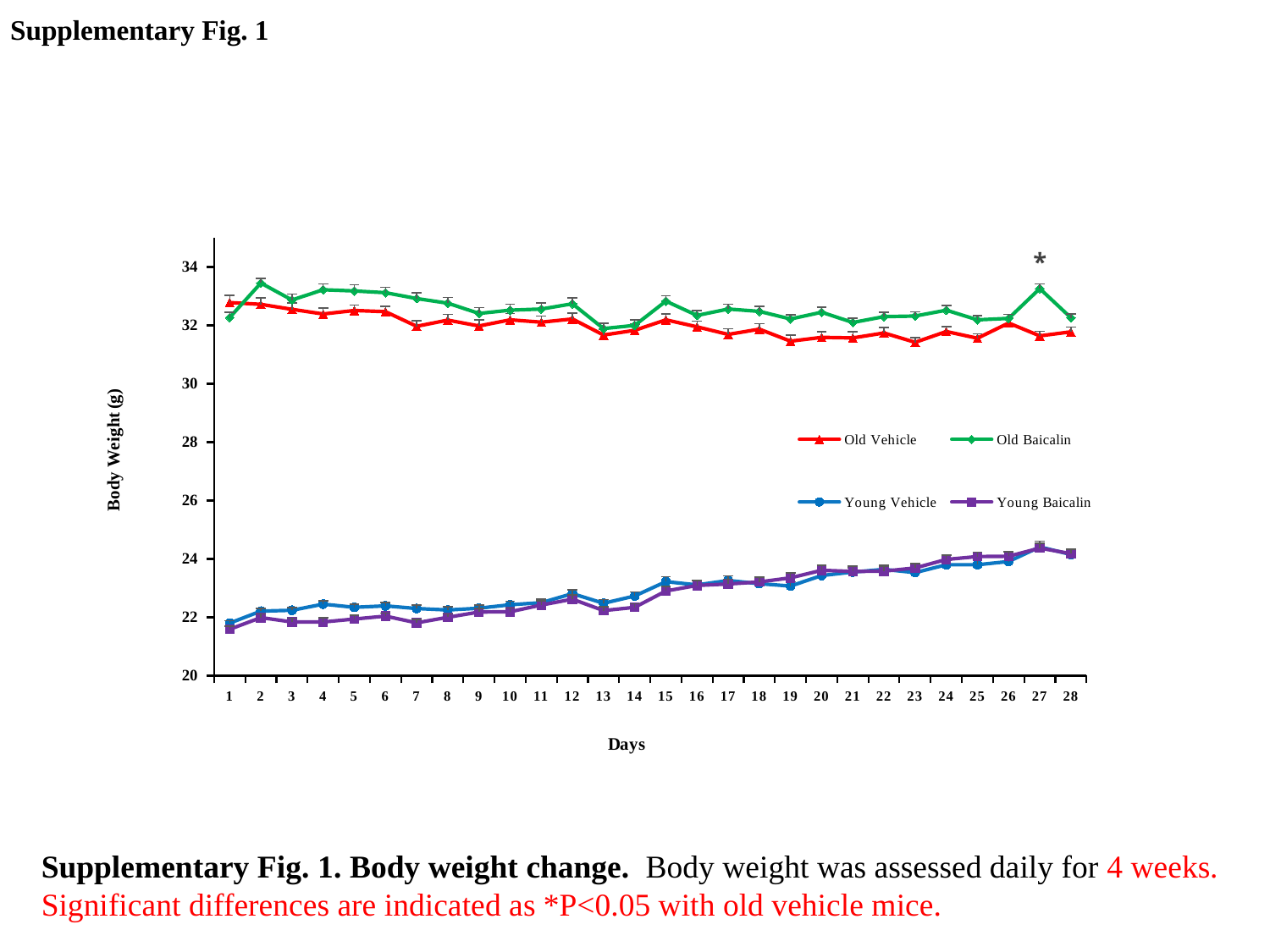

Supplementary Fig. 1
### Chart
| Category | Old Vehicle | Old Baicalin | Young Vehicle | Young Baicalin |
|---|---|---|---|---|
| 1 | 32.78 | 32.27 | 21.8 | 21.59 |
| 2 | 32.72 | 33.45 | 22.21 | 21.99 |
| 3 | 32.55 | 32.87 | 22.240000000000002 | 21.839999999999996 |
| 4 | 32.39 | 33.22 | 22.45 | 21.84 |
| 5 | 32.510000000000005 | 33.18000000000001 | 22.34 | 21.94 |
| 6 | 32.470000000000006 | 33.120000000000005 | 22.389999999999997 | 22.04 |
| 7 | 31.97 | 32.92 | 22.3 | 21.810000000000002 |
| 8 | 32.17999999999999 | 32.760000000000005 | 22.250000000000004 | 22.0 |
| 9 | 31.98 | 32.41 | 22.31 | 22.18 |
| 10 | 32.190000000000005 | 32.519999999999996 | 22.43 | 22.189999999999998 |
| 11 | 32.11 | 32.559999999999995 | 22.499999999999996 | 22.42 |
| 12 | 32.22 | 32.74 | 22.81 | 22.619999999999997 |
| 13 | 31.669999999999998 | 31.889999999999997 | 22.48 | 22.230000000000004 |
| 14 | 31.830000000000002 | 32.00000000000001 | 22.73 | 22.34 |
| 15 | 32.19 | 32.83 | 23.22 | 22.9 |
| 16 | 31.95 | 32.34 | 23.110000000000003 | 23.090000000000003 |
| 17 | 31.690000000000005 | 32.559999999999995 | 23.26 | 23.14 |
| 18 | 31.869999999999997 | 32.480000000000004 | 23.15 | 23.21 |
| 19 | 31.46 | 32.22 | 23.07 | 23.35 |
| 20 | 31.589999999999996 | 32.45 | 23.43 | 23.61 |
| 21 | 31.57 | 32.1 | 23.55 | 23.57 |
| 22 | 31.74 | 32.3 | 23.639999999999997 | 23.580000000000005 |
| 23 | 31.42 | 32.32 | 23.53 | 23.689999999999998 |
| 24 | 31.79 | 32.519999999999996 | 23.800000000000004 | 23.98 |
| 25 | 31.559999999999995 | 32.190000000000005 | 23.8 | 24.080000000000002 |
| 26 | 32.08 | 32.239999999999995 | 23.91 | 24.09 |
| 27 | 31.639999999999997 | 33.260000000000005 | 24.41 | 24.37 |
| 28 | 31.779999999999994 | 32.25999999999999 | 24.15 | 24.18 |Supplementary Fig. 1. Body weight change. Body weight was assessed daily for 4 weeks. Significant differences are indicated as *P<0.05 with old vehicle mice.
